# Antimicrobials and Antimicrobial Resistance in the Environment and Its Remediation: A Global One Health Perspective

**DOI:** 10.3390/ijerph16234614

**Published:** 2019-11-20

**Authors:** Ashok J. Tamhankar, Cecilia Stålsby Lundborg

**Affiliations:** 1 Global Health—Health Systems and Policy: Medicines, Focusing Antibiotics, Department of Public Health Sciences, Karolinska Institutet, 171 77 Stockholm, Sweden; Cecilia.Stalsby.Lundborg@ki.se; 2 Indian Initiative for Management of Antibiotic Resistance, 302, Aryans, Deonar farm road, Deonar, Mumbai 400088, India; 3 Department of Environmental Medicine, R.D. Gardi Medical College, Ujjain 456006, India; 4 School of Biotechnology, Kalinga Institute of Industrial Technology, Bhubaneswar 751024, India

## 1. Introduction

The awareness about pollution of the environment by antimicrobials/antibiotics is increasing globally. So is the literature, which is predominantly on antibiotic resistant bacteria, antibiotic resistance genes and antibiotic residues in the environment. The main concern about this, is the fear that resistance in the environment will get transferred to the clinical pathogens (for example, through horizontal gene transfer) leading to untreatable infectious diseases. It is estimated that antibiotic resistance may result in deaths of several million per year, if suitable measures are not taken up to mitigate the resistance problem [1]. The World Health Organization and the United Nations General Assembly have therefore called antimicrobial resistance a global threat that needs to be resolved with top priority [2,3].

The resistance generating sources in the environment are mainly human waste, animal waste and manufacturing waste. Both humans and animals (agriculture, poultry, aquaculture etc.), release large amounts of antimicrobials/antibiotics, which are consumed by them for therapeutic and prophylactic use, in the environment through excretions and improper disposal, and also the resistant bacteria in their systems, and make the environment prone to multiplication of resistant bacteria and abundance of resistance genes. An additional issue in this is the inappropriate use of antibiotics by humans for themselves and for their animals, because of lack of awareness regarding appropriate use of antibiotics. Interventions in the form of increasing public awareness and knowledge are the most commonly used strategies for effecting appropriate antimicrobial use and reducing antimicrobial resistance [4]. For example, in a survey in China it was found that the pig farmers’ knowledge regarding antibiotic use for their pigs was very poor and it was accompanied with improper behaviour. The survey results further showed that the probability of improper antibiotic use decreased with the increase in farmers’ knowledge about appropriate antibiotic use, and about the hazards of antibiotic residues in the environment [5]. The drug manufacturing units also, through their effluents, pollute the environment by antimicrobials. The available treatments/treatment plants for treating wastewater/effluents not being efficient to neutralize these pollutants, there is an abundance of antimicrobials/antibiotics, resistant microbes/bacteria and resistance genes in the environment. The share of literature is higher for antibiotic resistant bacteria compared to antibiotic residues and resistance genes as the detection of the latter two is relatively more expensive and also requires a little higher level of technology. In this article, we will mainly deal with antibiotic resistant bacteria, resistance genes and antibiotic residues in the environment.

## 2. Non-Aquatic Environment

Studies from several parts of the world have reported the presence of antibiotic residues, antibiotic resistant bacteria, as well as resistance genes in various non-aquatic environmental compartments such as soil, manure, animal meat, plants etc. [6,7,8,9,10]. In the context of resistance, the literature is more abundant for poultry, it being known as an extensive user of antimicrobials for growth promotion/prophylaxis besides the therapeutic use, and there are several reports of antibiotic residues, resistant bacteria and resistance genes being detected in poultry environment [11,12,13].

## 3. Aquatic Environment

Most of the antimicrobials/antibiotics used for various purposes and that from manufacturing plant effluents end up in the aquatic systems of the world environment, as well as the resistant bacteria and resistance genes generated by them. Thus, there are reports of their occurrence in hospital wastewater [14,15,16,17,18], rivers [19,20,21], rainwater-harvesting tanks [22], canal waters [23], recreational waters [24], municipal/community wastewater [25], and pharmaceutical plant effluent [26]. These wastewater discharges further have impact on various water bodies and contaminate them [27].

## 4. Non-Aquatic and Aquatic Environment Combined

While there are studies which look into only one of the many non-aquatic or aquatic compartments of the environment, there are many studies that cover both these types encompassing a composite environment. For example, water and plants [28], water and sand [24], wastewaters, natural and drinking waters and solid matrices such as sludge, sediment, and soil [8,29].

## 5. Resistance Built up in Bacteria after Exposure to Antibiotics in Environment

While in vitro studies show a link between antibiotic exposure and antibiotic resistance, experiments are also needed to be done in actual environmental niches to see whether resistance gets built up in the presence of antibiotics in an environmental compartment and whether antibiotic exposure causes any adverse effects on the environmental system. Two such experiments are cited here. In one experiment, in a turkey farm, it was found that resistance to enrofloxacin was detected at a very high frequency after treatments with enrofloxacin via drinking water, a representation of poultry drinking water from natural sources contaminated with antibiotic residues [30]. In another hydroponic experiment, representing plants growing in antibiotic contaminated waters, exposing pakchoi (*Brassica chinensis* L.) to antibiotic contaminated waters, resulted in detection of target antibiotics at concentrations ranging from 6.9 to 48.1 µg·kg^−1^ in the vegetable grown in contaminated water, and the rates of antibiotic-resistant endophytic bacteria as well as the resistance genes significantly increased in the plants [31].

## 6. Environmental Contamination by Antibiotics and One Health

We define here ‘One Health’ in the context of environment and antimicrobial resistance as, One Health is a study and interpretation of an integrated paradigm of antimicrobials and antimicrobial resistance dynamics and epidemiology, that encompasses human health, biodiversity health and ecosystem health including socio-behavioural aspects, that informs on the processes leading to the occurrence and recurrence of infectious agents and resistance and their dissemination and extinction in organic and inorganic habitats/environments, for the purpose of development of antimicrobial resistance management strategies. Few studies, projects or literature reviews encompassing all these dimensions for an organism or an antimicrobial in a particular niche/geographical area/ecosystem are evident in literature (e.g., [27,29]). Studies mostly occur in separate events and not as a conscious integrated event. In our project in India entitled ‘APRIAM-Studies on Antibiotic Use, Antibiotic Resistance and Antibiotic Residues in the Environment of India with a Context of Antibiotic Resistance Management in a One Health Approach’, we kept in focus a One Health approach while using varying study dimensions and while creating certain protocols [32,33]. Although the project is still ongoing, a mention of some of its results is worthwhile here to create a context between environmental antibiotic residues, antibiotic resistance, resistance genes and One Health. We found that, in people’s and healthcare worker’s perception, environment was intimately connected to occurrence of infectious diseases, antibiotic use and resistance development [34,35]; a time-series analysis study also showed that climatic factors influenced occurrence of Methicillin-Resistant *Staphylococcus aureus* (MRSA) skin and soft-tissue infections [36], and further we found that, resistance patterns were shared for *Escherichia coli* from humans, animal (cow) and their associated water when from an inland area, whereas, when located in the proximity of sea, resistance of *E. coli* from humans, animals and water had a shared pattern but it was different from the inland one [37]. We also found that in a niche area in a village, there was not only commonality of a resistance pattern of *E. coli* in humans, animals and the water in their environment but the commonality also extended to resistance genes [38]. In further exploration, we found that antibiotic residues, antibiotic resistance and resistance genes in water and sediments of a nearby river share some commonality [21]. As socio-behavioural and anthropogenic aspects also have an impact on the generation of resistance in the environment [39]. We also conducted studies on the same river about the impact of a special anthropogenic activity particular to India, holy dip and congregative holy dip of millions of persons in a holy river (Kumbh Mela) on antibiotic residues, antibiotic resistance and resistance genes (to be published). When our studies are complete, all these will be mapped from a One Health perspective.

## 7. Current Wastewater Treatment Failure

Wastewater is produced daily from various sectors and segments of society. Worldwide, 113 countries have data available on wastewater production, 103 countries on wastewater treatment, and62 countries on wastewater use [40] E ven after treatment, antibiotic residues, antibiotic resistant bacteria and resistance genes are still present in the wastewater, and the wastewater treatment plants (WWTP) are considered ‘hot spots’ of resistance multiplication [41] The wastewater from households, animal rearing facilities and WWTP effluents mostly get released into nearby waterways, wherefrom it might be used for irrigation purposes and studies have shown that some antibiotics have very long half-lives in agricultural soils: 55 to 578 days for tetracycline and 120 to 2310 days for ciprofloxacin [42,43,44]. Conventional wastewater treatment facilities typically have biological degradation, for example using the activated sludge process, whereas advanced facilities have tertiary treatment processes, such as reverse osmosis, ozonation, sonolysis and advanced oxidation technologies like fenton oxidation, heterogenous-photocatalysis with TiO2 etc. These treatments do not necessarily fully remove antibiotic residues, antibiotic resistant bacteria and resistance genes from the wastewater. For example, there are reports that antibiotic residues, antibiotic resistant bacteria and resistance genes still remain even after the conventional treatment [16,45]. Additionally, even after the advanced treatment processes currently in use, the problem is not fully resolved, for example, a study showed that even after ozonation treatment about 20% of sulfonamides, trimethoprim and macrolides still remained in the effluent [46].

## 8. Complete Remediation of Environmental Antibiotic Residues, Resistant Bacteria and Resistance Genes

Considering these issues and also that the normal photocatalysts used for disinfection are expensive materials like silver (Ag), titanium (Ti) etc., there was a need to develop photocatalysis based on inexpensive resources. Our research group has developed a technique using cheap resources like iron (Fe) or kaolinite nanoparticles and sunlight or visible light, that results in complete disinfection of multi-drug resistant pathogenic enteric bacteria and *Salmonella* from natural waters such as from ponds, rivers, lakes, tap water etc. [47,48,49].The same technique using the cheap resource of Fe and sunlight is also successful in 100% decontamination of environmentally highly stable antibiotics like ciprofloxacin from natural waters [50].This technique using sunlight is also useful with the conventional expensive photocatalysts [51]. Further, for this technique, we have been able to show that the genetic resistance material gets completely degraded by this technique and in the process, we have also developed an insight into how the resistance gets broken down [49,52].

## 9. Conclusions

There is a need for regulations to be established and implemented in many areas related to antimicrobials in the environment. The areas to focus are the pharmaceutical industry, hospitals, wastewater treatment plants, aquaculture farms, poultry farms, pig farms, and households. Other key areas to focus are strengthening and persevering awareness and education, antimicrobial stewardship strategies inclusive of environmental risk sensitization and management, pharmaceutical take-back programs, designing greener antimicrobials with better degradability in the environment, implementing environmental risk assessment prior to the launch of new drugs, monitoring release of antimicrobials into the environment, and eco-pharmacovigilance. The risk of using sewage/wastewater for irrigation needs to be carefully evaluated. Toxicological effects of antimicrobial use on non-target organisms and the environment should be addressed and informed to practitioners. There is a need to use less costly methods for antimicrobial residue measurements. Additionally, there should be methods of monitoring progress of correctives.

The whole gamut of antimicrobial/antibiotic use, antimicrobial/antibiotic residues, antimicrobial/antibiotic resistance, resistance genes, and horizontal gene transfer is interconnected, one leading to another and finally resulting in increased antimicrobial/antibiotic use, which further leads to the same consequences. Therefore, there is a need to develop and implement instruments to carefully monitor antimicrobial/antibiotic use in community, animals, and hospitals, as well as residues, resistant microbes/bacteria and resistance genes in all compartments of the environment, and to update this information on a continuous basis. The crisis of antimicrobial/antibiotic resistance is reaching unmanageable proportions and if immediate measures are not taken to resolve the problem, simple infections may become life threatening.
